# Mechanism Models of the Conventional and Advanced Methods of Construction Safety Training. Is the Traditional Method of Safety Training Sufficient?

**DOI:** 10.3390/ijerph20021466

**Published:** 2023-01-13

**Authors:** Aminu Darda’u Rafindadi, Nasir Shafiq, Idris Othman, Miljan Mikić

**Affiliations:** 1 Department of Civil & Environmental Engineering, Universiti Teknologi PETRONAS, Seri Iskandar 32610, Malaysia; 2 Department of Civil Engineering, Faculty of Engineering, Bayero University, Kano P.M.B 3011, Nigeria; 3 Department of Engineering Management, Faculty of Engineering, University of Leeds, Leeds LS2 9JT, UK

**Keywords:** mechanism model, cognitive psychology, cognitive learning theory, the conventional method of safety training, the advanced method of safety training, construction industry

## Abstract

Cognitive failures at the information acquiring (safety training), comprehension, or application stages led to near-miss or accidents on-site. The previous studies rarely considered the cognitive processes of two different kinds of construction safety training. Cognitive processes are a series of chemical and electrical brain impulses that allow you to perceive your surroundings and acquire knowledge. Additionally, their attention was more inclined toward the worker’s behavior during hazard identification on-site while on duty. A study is proposed to fill the knowledge gap by developing the mechanism models of the two safety training approaches. The mechanism models were developed based on cognitive psychology and Bloom’s taxonomy and six steps of cognitive learning theory. A worker’s safety training is vital in acquiring, storing, retrieving, and utilizing the appropriate information for hazard identification on-site. It is assumed that those trained by advanced techniques may quickly identify and avoid hazards on construction sites because of the fundamental nature of the training, and when they come across threats, they may promptly use their working memory and prevent them, especially for more complex projects. The main benefit of making such a model, from a cognitive point of view, is that it can help us learn more about the mental processes of two different types of construction safety training, and it can also help us come up with specific management suggestions to make up for the approaches’ flaws. Future research will concentrate on the organizational aspects and other cognitive failures that could lead to accidents.

## 1. Introduction

Because of its dynamic and unique nature, construction is one of the most dangerous industries globally, experiencing many accidents. Despite giving safety training to workers regularly, the accident rate increases around the globe, and Malaysia is no exception. Construction workers are more likely to be involved in fatal accidents than workers in other businesses, and their death rate is nearly triple that of other industries [1]. The incident could be attributable to inadequate safety training practices and occupational hazard identification. Construction accidents are primarily due to insufficient safety education, training, and job hazard identification and control [2]. A significant source of concern in safety management is a lack of adequate worker construction safety training and job hazard identification [3]. Workers who are not adequately trained are less capable of recognizing potentially hazardous situations on-site [4]. Workers’ willingness to adopt safe work practices is also heavily influenced by safety perception, the level of safety education, and the training they have received, to mention a few [5]. Safety training teaches workers about safe work practices and reduces accidents’ frequency and severity on construction sites.

The construction workers are trained traditionally (2D drawings, pictures, or photographs) or using advanced safety training methods (visualization technologies). The construction industry needs to improve the existing paper-based and manual safety processes [6]. Conventional safety training methods in the construction industry are inefficient [7]. These inefficiencies include the following. Job hazard identification is made by the relevant professionals’ expertise, based on 2D drawings, pictures, or static drawings, and thus might never be detected, even by experts. Revising the safety plan every time the construction schedule or design changes is time-consuming and challenging. Foreign workers are not primarily given safety training in their mother tongue or in a way that considers their literacy level [8]. Another major factor affecting construction safety training and job hazard identification is the language barrier between the instructors and the laborers or co-workers [9]. A more significant number of construction accidents occur due to the differences in language between the local and foreign workers [10]. Because Spanish is their primary language, many Hispanic workers in the United States construction industry struggle to understand their supervisors’ safety instructions, leading to poor safety performance and low productivity [11].

According to the study, 3D simulated virtual job sites effectively enhance learning in construction workers with limited English proficiency during training sessions [9]. Loosemore and Malouf [12] also recommended using new interactive and immersive technologies for construction safety training to create a strong connection between the trainees and the subject matter. Using innovative training practices for construction workers is one of the most effective ways to improve safety performance. Because of the growing number of foreign construction workers in international construction projects, innovative safety training and job hazard identification methods must compensate for the ineffectiveness of traditional counterfeits. BIM-based tools can establish a structure’s virtual construction before or during construction to reduce uncertainty, ensure safety, identify issues (collision detection), and simulate and analyze potential dangers [13,14]. The advantages and disadvantages of advanced and conventional safety training methods and job hazard identification affect construction employees’ cognitive capacities to learn and apply proper safety knowledge to recognize and avoid near-misses or accidents on-site. Here are some reviewed studies about cognitive models and the limitations that led to this research.

Reason [15] proposed a human error-based cognitive modeling system focusing on skill-based, rule-based, and knowledge-based errors. Mitropoulos et al. [16] built a cognitive model of construction safety. They found that the work requirements and skills impact the likelihood of error and accident. A human general information processing model was presented by Wickens et al. [17]. This model comprises four cognitive stages—sensory processing, perception, response selection, and response execution—and three functional modules—attention resources, working memory, and long-term memory. Human mistake is the leading cause of mining accidents in India, according to Mohan and Duarte [18], who developed a cognitive model to investigate these occurrences. The model considered both internal and external influences, consisting of five key stages: sense transducer, integration, decision-making, response selection, and response execution. Nakayasu et al. [19] proposed a simple human cognitive reliability model consisting of perception, cognition, and action processes to examine human driving faults by evaluating performance based on response time.

Deng et al. [20] developed a cognitive failure model based on cognitive safety theory and cognitive factors to describe the causes of construction workers’ unsafe behaviors. The study concluded that there are five cognitive link failure factors: safety vigilance, hazard recognition, safety knowledge, safety behavior attitude, and professional skills. However, there was no strong correlation between safety knowledge and expertise and the other three cognitive factors. This suggests that other cognitive failure variables may be the primary cause of cognitive failure in comprehending the information and taking action. The cognitive factors that lead to risky behavior are: not being aware of the hazard, not knowing the danger, choosing unsafe behavior, and being unable to avoid the risk [21]. Shi et al. [22] developed a model of miners’ risky conduct intentions, based on the theory of planned behavior (TPB), with three cognitive structure variables representing risk perception, behavior experience, and safety consciousness. They discovered that cognitive factors significantly affect miners’ intentions to engage in dangerous behavior. Goh et al. [23] evaluated the relative impact of distinct cognitive elements within the theory of reasoned action (TRA), formerly known as TPB, in determining safety behavior using a supervised learning approach. Intention and social standards were determined to have the most significant impact on whether a worker was observed to be working safely or not. Liang et al. [24] conducted a study to determine how stress influences risky behavior. The final stress–cognition–safety model for construction workers confirmed that physical and emotional stress affects safety awareness, safety knowledge, subjective norms, safety attitude, and perceived behavioral control. In addition, they discovered that stress indirectly influences the unsafe conduct of construction workers via three cognitive components (safety awareness, subjective norm, and safety attitude) and that both physical and emotional stress directly cause dangerous behavior.

Fang et al. [25] developed a cognitive model of construction workers’ risky behavior based on cognitive and social psychology theories and existing accident causation models. They argued that the cognitive process could be separated into five connectors: information acquisition, information comprehension, perception of response, response selection, and action. The model methodically and exhaustively explains the mechanics of unsafe behaviors from an individual cognitive perspective and can explain the mechanisms by which internal cognitive elements and external organizational factors lead to risky behaviors among construction workers. However, the cognitive model is still at the level of a theoretical framework and lacks the cognitive aspects necessary to describe it. Choi and Lee [26] developed an agent-based model that combined theoretical and empirical evidence regarding socio-cognitive processes. The model was utilized to evaluate how the socio-cognitive process interacts with safety management interventions and affects workers’ safety behaviors in low-, moderate-, and high-risk environments. The findings revealed that all three interventions contributed to reducing the incidence rate. Nevertheless, the model assumes that the site risk does not change during the simulation, even if workers are exposed to varying degrees of the actual danger, based on the beta distribution given by the static site risk value. Ye et al. [27] built an agent-based modeling (ABM) technique for examining construction workers’ socio-cognitive processes, in the context of interactions with foremen, managers, and co-workers. The simulation results indicate that the effects of three social groupings on workers varied. Safety demonstration is frequently more successful than safety communication in preventing risky behavior among workers by the foremen. The influence of manager behavior feedback and safety training on risky worker behaviour is substantial, but excessive safety training is ineffective. The influence of coworkers on the safety habits of workers is not always good. However, the influence of safety training intensity on the risky behaviours of workers was not evaluated in this study.

Most of the abovementioned scholars have studied the influencing factors of unsafe behaviors from the cognition perspective. They discovered that workers’ unsafe behaviors are due to the abovementioned cognitive factors. However, most of these factors, if not all, are usually due to a lack of adequate safety training. Safety training is crucial because it raises employee awareness, boosts worker morale, reduces the risk of accidents and injuries, boosts productivity, fosters communication, and prevents the monetary expenses of accidents and occupational illness. The models presented provide a foundation for developing a cognitive model based on workers’ behaviors, but not obtaining safety information (i.e., safety training). The previous studies rarely consider the cognitive processes of two kinds of construction safety training. Cognitive processes are a series of chemical and electrical brain impulses that allow you to perceive your surroundings and acquire knowledge. Additionally, their attention was more inclined toward the worker’s behavior during hazard identification on-site while on duty. A study is proposed to fill the knowledge gap by developing the mechanism models of the conventional and advanced methods of construction safety training, based on cognitive psychology and cognitive learning theory. The paper is structured as follows: the next section provides the background of the study, Section 3 presents the mechanism model, and the last section provides the conclusions.

## 2. Background of the Study

Problem solving is a higher-order cognitive activity and intellectual function that necessitates regulating and controlling more basic or everyday talents. There are two types of problems: well-defined knowledge-rich problems and ill-defined knowledge-lean problems [28]. The former is the expected kind of problem, and the potential solutions are known and already in place to solve it, while the latter is the kind of unexpected problem, and the possible solutions are unknown. Most of the safety issues (hazards) are well-defined [29]. That is why construction workers are trained to identify and avoid near-miss or accidents while on duty on-site. Well-defined issues are preferred because they can be executed, have an ideal strategy for the solution, have an objectively standard solution, and assess strategy faults and weaknesses. However, there could also be ill-defined knowledge-lean problems on-site due to the project’s complexity, the kind of safety training construction workers receive, and other relevant factors. In that case, the experts must intervene to determine the appropriate solutions to the encountered problems because there are no standard solutions. The following sub-sections explored some problem solving theories and how they could apply to construction safety.

### 2.1. Behaviorism

Behaviorism, also known as behavioral psychology, is a theory about learning that asserts that all behaviors are acquired by interacting with the environment via a conditioning process [30]. It is focused exclusively on observable external stimulus-response behaviors that can be investigated systematically and visibly. Conditioning can be classified into two broad categories in behavioral psychology: classical and operant conditioning. However, operant conditioning is usually applied on construction sites. It is a form of reinforcement- and punishment-based learning, also known as instrumental conditioning [31,32]. It establishes a link between a behavior and its associated result. For instance, the management should reward safety-abiding construction workers, and those violating safety should be penalized or punished. The workers’ behavior may change because of the outcome due to safety violations. When the desired effect occurs due to an activity, the behavior becomes more likely to repeat itself in the future and vice versa [33]. According to social cognition theory, new employees will replicate activities they observe others performing, followed by a reward [34]. This theory is utilized mainly by children and animals but can monitor safety violations on construction sites. Any construction worker, regardless of background, can be trained to abide by the safety rules and regulations with proper conditioning, as strict behaviorists believe that all actions are reproducible because of experience.

The theory is not without limitations and is explained as follows. It is based on a trial-and-error approach and without organized methodology. Repeated and varied attempts are the characteristics of this type of behavior. Behaviorism theory is not systematic and does rely on any idea, insight, or organized methodology. Hazards are generally identified through organized procedures on-site because of the safety training received by the workers before construction projects start, not by trial-and-error learning. It might be tedious and time-consuming to locate hazards on-site without adequate and proper safety training or knowledge and could lead to near-misses or fatal accidents on-site. It is potentially risky, especially when trying to find job hazards through a trial-and-error approach, and thus could lead to a deadly accident. Therefore, safety training cannot be based on this learning theory because of the constraints mentioned above, but can be used to monitor the safety violations by the construction workers on-site.

### 2.2. Gestalt Psychology

According to the Gestalt problem solving theory, problems are solved because a flash of insight (representation or restructuring) a problem solver creates facilitates the solution [35]. Insight can be conceptualized in various ways, depending on the area of application. Mainly in the construction industry, it involves finding problems such as what construction workers were shown or taught during the safety training. Gestalt psychologists believe that solving an issue makes it complete and eliminates the problem’s incompleteness [36]. The closure of a problem is usually accompanied by an insight or ‘aha’ moment. When people become instantly aware of the solution to a problem during constructive thinking, this is called insight. According to Gestalt psychologists, problem solving is a productive process. Barriers to problem solving are usually mental set or functional fixedness. Mental set arises when a problem solver becomes obsessed on employing an approach that previously worked, but is no longer effective in solving the current situation [37]. When a problem solver fails to understand that objects can be utilized for purposes other than those for which they were designed, functional fixedness occurs [38].

Most of the hazards or safety problems on construction sites do not usually require representation or restructuring before they can be solved because of the safety training received before the commencement of the project. For example, fall protection is needed for any task of about 1.8 m above the ground; a slab edge requires a guard or handrail to prevent falls from height, and a hole or opening more than 50 mm requires a cover to avoid the leg from being stuck or falling objects from the higher floors. However, there might be some safety issues that could arise on-site that may require using insight by mental representation or restructuring the problem to find a solution. Gestalt psychology refers to this process of solving a problem through learning or experiencing ‘functional fixedness’. This theory believes that humans and animals have some built-in understanding that assists them in solving problems through mental representation or restructuring. However, because of the complex nature of construction projects, insight is not enough to identify hazards on-site without proper and adequate safety training. Additionally, functional fixedness cannot be relied upon because the subsequent project might differ from the completed one. The theory does have some limitations. Gestalt psychology made no explicit reference to the phenomena of problem solving representation or restructuring, functional fixity, or insight. Additionally, this approach lacked theories, models, and procedures for restructuring the problem.

### 2.3. Representational Change Theory

The representational change theory integrates Gestalt concepts into a functional approach with explanations [39]. This theory is often referred to as thinking outside the box. Representational change can occur in an individual’s mind because of the addition of new information (elaboration), rules reinterpretation (constraint relaxation), and if functional fixedness is eliminated (re-coding). A problem is conceptualized in the mind of a problem solver to identify the knowledge stored in long-term memory. The retrieval mechanism distributes activation across ‘significant’ long-term memory storage. A block occurs if the information retrieved from the long-term memory search does not help solve a problem [40]. Insight problems result in impasses, leading problem solvers to develop ineffective initial representations [39]. Insight is attained when a barrier is broken (initial representation is changed) and knowledge is retrieved. The way the problem is represented changes, and the memory search is expanded, resulting in the availability of new knowledge. Understanding comes when self-imposed constraints on an issue are relaxed, deconstructing the problem into more minor elements [41].

Insight problem solving positively correlates with recognizing previously presented things in the working memory [42]. The connection between working memory and insight problem solving may shift [43]. Working memory, in contrast to long-term memory, which is the massive amount of knowledge kept throughout one’s life, is the tiny quantity of information retained in the mind and used to conduct cognitive actions [44]. Non-insight problems are usually less complex, while insight problems are more complex, which causes an impasse, for example, most of the time. While working memory is required for insight problem solving, it is not necessary to the same extent as for non-insight problem solving [45]. Under multitasking conditions, both insight and non-insight problem solving methods deteriorate [46]. While non-insight problems are related mainly to the working memory control system, insight problems relate to relevant storage systems (long-term memory) [45]. The insight process necessitates using many functional memory systems throughout the solution. Working memory’s significance in insight problem solving originates from information-processing theories that consider insight as a possible representational alteration within working memory [39]. Working memory capacity strongly correlates with problem solving ability and creativity [47].

Non-insight problems are designed to be solved through the systematic application of knowledge and logical reasoning, whereas insight problems need the problem solver to alter his perspective and approach the issue in a novel way to reach the solution [48]. Wearing personal protective equipment (PPE) does require insight and basic problem solving skills or abilities and is a perfect example in the construction industry. Using personal fall arrest systems (PFAS) requires insight and basic safety training to avoid an on-site accident. PFAS is used by connecting it to a harness to secure an anchor device tied to a structural element and a shock-absorbing connecting assembly designed to mitigate the wearer’s kinetic energy falling to the ground, for example, if the structural part or member intended for this task does not have the required strength (incapable of bearing at least 5000 pounds (22.2 kN) for each employee attached, for example), the need for a substitute or representational change may be required of the initial plan. A structural member capable of withstanding the calculated weight must be identified and used to avoid fatal accidents on-site. Insight plus safety training may be enough to identify hazards on-site, but not necessarily enough to require a representational change to mitigate or eliminate the accident. However, some safety issues might require representational modification before solving them in the construction industry. If safety rules are reinterpreted (constraint relaxation) whenever there is a need to solve a particular problem, there are bound to be many constant safety violations. What specific issues need representation must also be categorically stated to avoid confusion and safety violations and maintain the hierarchy of work procedures. Because of the complex and dynamic nature of the construction processes, many factors could affect finding solutions to insight problems on the construction site.

### 2.4. Information Processing Approach

The theory postulates that problem solving behavior is generated by a small number of simple information processes stored and arranged in the human brain. The approach also proposes that we search the problem space for the solution. The term “problem space” refers to the complete set of components involved in resolving an issue [49]. The process begins by defining the problem scope, identifying and testing possible solutions, and selecting and implementing the appropriate solution. The problem space comprises two states: the initial state, i.e, the current situation, and the objective state, or the solution, which we attempt to arrive at [50]. The greater the distance between two states, the more complicated the problem space becomes. Each state in a problem space can be viewed as a potential state of knowledge that the solver could acquire. A state of knowledge is simply what the solver knows about the problem at any given point in time, learning in the sense that the information is readily available and retrievable in a fraction of a second. This approach works well for well-defined problems, but not for ill-defined issues [51]. The actions we take to move from one state to another are operators. Operators are the permissible operations performed within the problem space to produce a solution [52]. The problem space’s structure defines the good moves and the direction of movement toward or away from the objective, and it interacts with the constraints of short-term memory to make some solution paths simpler to locate than others [53]. The problem space and task environment are inextricably linked. A task environment is a theoretical construct that provides the goal-relevant implications of permissible task movements [54]. The relative ease with which a problem can be solved is contingent upon the solver’s ability to accurately represent the essential aspects of the task environment in his problem space. The quest for a solution is an exploration through the problem space, from one knowledge state to the next, until the current level of knowledge encompasses the solution.

The structure of the problem has a significant impact on the search behavior of the information-processing system, and this influence is a direct result of the system’s limited short-term memory capacity. The field of information processing is particularly interested in how individuals choose, store, and recall memories. A person’s short-term memory capacity is limited to seven items, plus or minus two [55]. However, some scholars argue that the number may be smaller. The human information-processing system operates as a serial system [56]. No behavior of problem solvers necessitates the simultaneous rapid search of disparate sections of the problem space. Sequentially generating information states to create one that satisfies the problem objectives is the search strategy [51]. Heuristic strategies search for the ideal solution to minimize search time and cognitive stress. A heuristic is a process that allows us to discover something new and acts as a cognitively undemanding strategy that often produces a solution, but not necessarily the optimal one [57]. They function within the human processing system and can solve well-defined problems. These strategies include, but are not limited to, the trial-and-error approach (one solves a problem by randomly attempting solutions, until one reaches the desired result), hill climbing (a problem is solved incrementally toward the broader aim or task), means–ends analysis (an issue is solved by considering the impediments that exist between the initial problem state and the desired state, and the problem solver then establishes subgoals for removing each of these impediments), and analogy (one solves an issue by referencing the solution of a previously solved problem). The information processing approach leads to successful computer models in the construction industry and other fields. This theory does not perform well on insight problems, and most everyday problems are ill-defined ones, and construction sites are no exception. Additionally, it is often impossible to first generate and hold the entire problem space in mind and then only search for the optimal solution.

A safety management officer received a complaint that those working on the scaffold unintentionally allowed the fall of tools and other objects to the lower floors. Falling tools or things can lead to a fatal struck-by accidents. Lack of safety nets, workers’ tool belts, and scaffold toe boards cause this type of accident on construction sites. Then, the safety officer and site supervisor/manager would inspect the site and observe and take note of the seriousness of the situation. In this example, the problem does not require insight because the factors responsible for the falling objects are visible, even to a non-expert. The probable questions that would come to the mind of the safety officer could be the following to solve the problem. Are the remedies (preventive measures) available on-site? If not, then are they provided in the working bill? If yes, they should be purchased, equipped, and installed to mitigate or prevent accidents. If not, they should be added to the contingencies and source for the fund to cover and solve the problem. The initial and final step taken to resolve the safety issue is the problem space, and the actions taken along the line are the operators. Direct instruction was used as the heuristic to solve the identified problem.

### 2.5. Analogical Problem Solving

The theory of analogical problem solving could generate approaches in other domains of cognition [58]. The basic idea behind analogical reasoning is to apply the same procedure as the previously solved problem to solve a similar situation. Problem solving is a sophisticated cognitive process that entails the creation of action sequences to accomplish a specific objective [59]. The method of analogical problem solving is conceptualized in three main stages: matching, mapping, and application. The first step is recognizing and checking that the information stored in the memory is relevant to solving a target problem. It entails locating an analogy in the long-term memory and adapting it to the current situation. A better grasp of how analogies are retrieved and recognized is necessary to successfully teach analogies as a problem solving heuristic method [60]. The mapping process involved in analogy utilization may contribute to several cognitive skills. Analogizing entails a methodical mapping of a hierarchical system of relations from one domain to another on a one-to-one basis. They are also used to map the representations of two or more occurrences of the solved problem onto a new one encountered. Meaningful parallels between problems can be identified more quickly if they share a high degree of similarity on the surface or in terms of deep structure [59]. Analogical problem solving could be a transformational analogy or a derivational analogy [59]. The first is concerned with direct solution transfer, whereas the latter is concerned with generating a new solution based on previously adapted reasoning processes. The derivational analogy strategy requires a more sophisticated and systematic approach to problem solving than the more straightforward transformational strategy, which relies on a simple matching procedure. The solution is slightly tweaked to match the new situation in a transformational analogy. Past reasoning processes are applied and altered in derivational analogies to arrive at a likely unique solution.

Individuals who received extensive training are more likely to employ a transformational analogy, whereas those who received insufficient training may use a derivational analogy [59,61]. The difficulty in adopting analogical problem solving is also a function of the solver’s capacity to transform prior solutions to help solve the new problem when required in long-term memory [62]. Solving diverse situations that require comparable solutions is a critical ability in everyday life, and the construction sector is no exception. Analogical problem solving entails manipulating a solver’s mental representation of a preceding problem. Novices solve problems primarily through imitation, a suboptimal kind of analogical problem solving [62]. They frequently see the resemblance between situations, in terms of their surface characteristics, rather than a profound representation of an earlier solved problem in the memory.

This theory may be suitable for construction safety training for both experienced and newly employed workers, especially for well-defined knowledge-rich problems using visual presentations (e.g., virtual reality (VR) or augmented reality (AR)). It is because most of the possible hazards can be identified before the commencement of the project using advanced visualization technologies. Trainees learned better using a close-to-reality, virtual, three-dimensional environment than those trained using traditional, for example, crane dismantling procedures [63]. However, ill-defined knowledge-lean issues may be only suitable for experienced personnel. Experience is the key to using an analogical problem solving approach. Visual presentations or imageability can be beneficial if they accurately depict the problem-solution relationship and facilitate analogical reasoning, possibly serving as a recall cue [64]. In our terminology, a well-defined knowledge-rich problem is when construction workers receive safety training using the exact drawings they would use during construction projects. While the ill-defined knowledge-lean problem is when construction workers receive safety training using the different drawings than they would use during real construction projects. In both scenarios, the workers require insight to solve safety issues on-site, but the latter may require creative insight to solve a problem. During the training, the workers are shown all the potential hazards on-site, and they can avoid them.

### 2.6. Summary of the Theories

Based on the problem solving theories explored, the following conclusions were made. Behaviorism theory can monitor safety violations on construction sites based on reward and punishment. Most of the hazards or safety problems on construction sites do not usually require representation or restructuring before they can be solved because of the safety training received before the commencement of the project. However, insight is needed to solve some safety issues, but is inadequate without safety training. Representational change theory, also known as thinking outside the box, may not be suitable for construction safety because it will bring safety violations and changes in the hierarchy of work procedures. There is always new information, rules reinterpretation, and re-coding. The information processing approach of problem solving may be the best for construction safety training, but only if a ‘direct instruction (laid down rules and regulations)’ heuristic is used. Workers must remember the dos and don’ts on construction sites to avoid near-miss or accidents. Analogical theory of problem solving may also be the best for construction safety training. The basic idea behind analogical reasoning is to apply the same procedure as the previously solved problem to solve similar situations or scenarios that could be encountered on-site. Construction workers could use the same thing they learned during safety training on-site in the actual project execution. Thus, by combining the information processing approach and analogical theory of problem solving, construction workers may be provided with befitting safety training using the advanced method.

## 3. Mechanism Model

The strengths (e.g., job hazard identification is straightforward, single integrated model, both static and dynamic environments are observable, knowledge management purpose) and weaknesses (e.g., human error, safety training and problems are implicit, use of static drawings or pictures, time-consuming, organizational culture) of the advanced and conventional safety training method affect the cognitive abilities of construction workers to learn and apply the appropriate safety knowledge to identify and avoid near-misses or accidents on-site. Cognitive failures occur when personnel are trained to utilize the old technique due to inefficiencies. Inadequate safety training is a contributing factor to unsafe worker behaviour [8]. Safety training educates employees on safe work procedures and minimizes the incidence and severity of accidents on construction sites. Training promotes safe surroundings, attitudes, and employee conduct [65,66]. The risk-taking propensity of employees is impacted by their safety training and supervision [67]. The propensity of workers to embrace safe work practices is also significantly influenced by safety perception, quality of safety education, and training, among other factors [5]. Conventional hazard recognition systems have not adequately addressed the issue of low-hazard recognition systems [68]. New safety training methods can help workers recognize hazards [69]. Workers trained with 3D BIM simulation demonstrated a greater comprehension of safety training than those trained conventionally [70]. Workers can rapidly assess and identify potential dangers in a visual environment, thus enhancing and accelerating their safety training [71].

### 3.1. Model Development

This part focuses on the theoretical evolution of the model. This model describes construction workers’ cognitive processes during and after safety training. Safety training refers to workers’ frequency, effectiveness, and thorough training to avoid accidents [72]. The safety training is categorized into conventional and advanced methods in this study. Workers are trained to identify the potential hazards and prevent them on-site (i.e., prevent an accident). Cognitive research examines learning mechanisms and how knowledge and skill are applied to various simple-to-complicated construction activities. Cognitive psychology is concerned with humans’ perception, attention, memory, mental, language, and decision-making processes [73]. The study adopted and used Bloom’s taxonomy of cognitive learning theory [74]. Cognitive learning involves using facts, rules, principles, and procedures and is broken into six progressive steps. They include obtaining information (relevant safety knowledge), understanding and processing information (i.e., answering ‘what’ and ‘why’ questions regarding the knowledge obtained), application, analysis, synthesis, and evaluation. Once the model is applied in an organization, it will create a safety knowledge management system and culture, where the three upper Bloom’s taxonomy levels will be utilized to evaluate and predict hazards.

**Figure 1:**
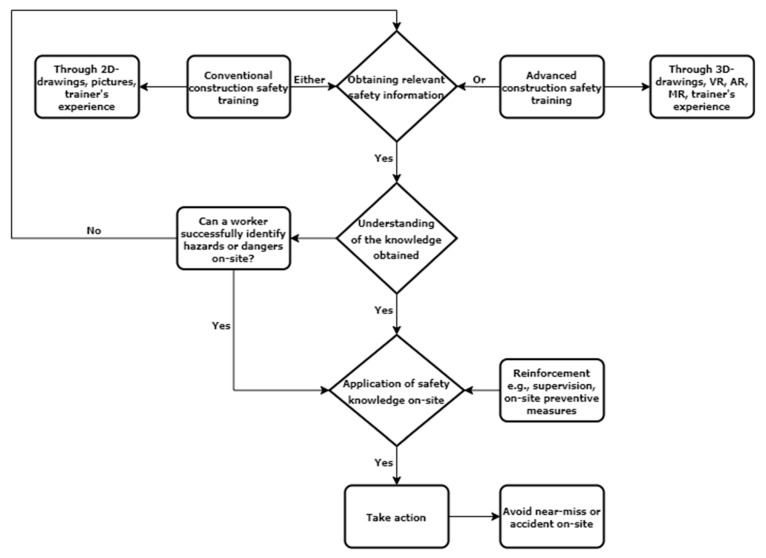
Cognitive processes of construction safety training.

The analysis assumes that construction workers who received advanced safety training can quickly identify and avoid hazards on-site. The model also assumes that cognitive failure at any stage leads to near-misses or accidents on-site. In contrast to other industries, construction workers do not have the opportunity to rectify their errors [25]. Near-misses or accidents usually happen due to workers’ unsafe behavior and lack of appropriate safety training. If workers engage in risky actions, their cognitive processes must be an issue [75]. Many risks in the construction industry are evident and straightforward to identify. When provided with the proper safety training, workers generally identify hazards using their eyes or ears and apply the necessary actions to prevent accidents [25]. The mind’s cognitive architecture is the information processing system that governs the flow of information and how it is acquired, stored, represented, altered, and accessed. Based on the reviewed past studies in the introduction section, the cognitive processes were generally found to be: obtaining information, understanding information, perceiving a response, selecting a response, and taking action. However, due to the nature of construction safety training, this study adopted the same, but the last three were merged as one (i.e., application). Their combination is the application of safety knowledge on-site to avoid near-misses or accidents. From the perspective of information processing, cognitive processes comprise three stages: receiving, storing, and utilizing information. The cognitive processes of the construction safety methods are shown in Figure 1.

#### 3.1.1. Obtaining Information

This stage entails personnel gaining the appropriate safety knowledge to avoid potential hazards on-site through traditional or advanced safety training. Safety training can help increase workers’ safety knowledge and awareness [76]. Additionally, it has been demonstrated to be a valuable source of knowledge for identifying potential hazards and preventing on-site accidents [77]. The knowledge obtained from safety training (external environment) through the sensory memory usually enters the working memory. The cognitive process employs a two-step method to ensure the quantity and quality of information entering working memory from the external environment. The outer filter is the first step, as it operates before the information is seen or heard by sense organs [78]. If the information is gathered deliberately, it will be transferred directly to working memory for processing. Otherwise, it will go through the second filter, known as the internal filter. An internal filter is where a person selects or discards the information captured unintentionally in working memory before it goes to long-term memory [78]. Then, the information gathered will then enter the working memory. Working memory is a conscious system that receives data from the memory buffers connected with different sensory systems [79]. The chunk of long-term memory is active, where workers’ cognitive activities predominantly occur.

Additionally, it organizes all incoming data, eliminates redundant data (filtering), and forwards the processing result to the next mental stage. What matters most in the learning settings is not one’s working memory capacity, although it is a factor in processing speed, i.e., one’s ability to evoke knowledge stored in long-term memory and apply it to the present situation. Although working memory does not directly access the data stored in long-term memory, it can retrieve data by changing calling conditions or evoking stimuli. “Similarity-matching” refers to the process through which experience or information that corresponds well to the calling conditions is quickly retrieved [80]. Frequency gambling regulates the amount of data entering working memory by similarity matching [81]. For instance, during any task operation on-site, when a construction worker encounters a potential hazard, working memory generates calling conditions that automatically cause the long-term memory to access the associated safety knowledge. However, this occurs if a worker is aware of what to do. Workers develop a detailed cognitive response and evaluate the risk, in order to interpret the risk information into problems or objectives [82]. The mental process may fail if the calling conditions cannot extract the appropriate information from the long-term memory using similarity matching and frequency gambling [25]. When a worker comes across a hazard he does not know about, that leads to non-intentional searching for relevant information. When confronted with truly novel challenges, construction workers, for example, revert to their repertory of ineffective solutions [83]. A near-miss or accident may occur on-site once there is a cognitive failure to retrieve and use the relevant safety knowledge. The nature and organization of cognitive content are critical for understanding how people answer questions and solve problems and how they differ, in this regard, as a function of the conditions of instruction and learning [84]. These cognitive characteristics, limited rationality, and reluctance in rationality show that workers cannot be entirely rational; thus, mental defects cannot be eliminated [25].

**Figure 2:**
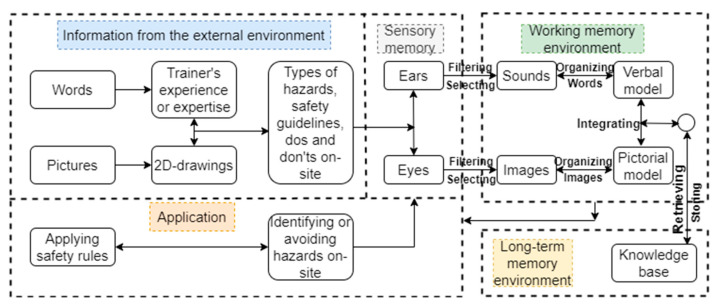
An information processing model of the conventional method of safety training.

**Figure 3:**
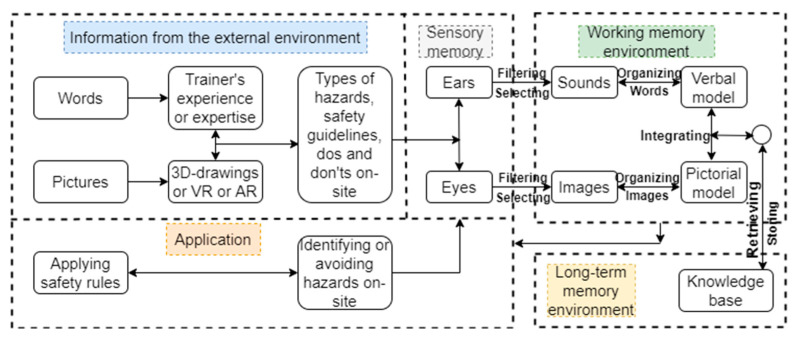
An information processing model of the advanced method of safety training.

The construction site is complex; workers require safety training to work safely without accidents. The cognitive process starts with obtaining the appropriate safety training for job hazard identification. If construction workers receive the proper safety training to identify potential hazards before the commencement of any project, near misses or accidents are less likely to occur. With a limited understanding of safety, untrained personnel may not spot an encircling hazard and fail to recognize the risk [72]. They may not seek dangers on purpose or may be incapable of detecting threats while searching inadvertently [85]. An information processing model of the conventional safety training is shown in Figure 2, and that of the advanced approach is presented in Figure 3. The only difference between the two is that the conventional method is based on two-dimensional drawings or pictures, while the advanced one could be based on virtual reality (VR), augmented reality (AR), or mixed reality (MR).

#### 3.1.2. Comprehension of the Knowledge Obtained

Safety knowledge comprehension is the ability of workers to understand and grasp the information given to them using either the traditional or advanced methods. Lack of proper understanding of the safety training may lead to cognitive failures and near-misses or accidents on-site. After the working memory has gathered the necessary information, the cognitive process advances to the second stage of information comprehension. Workers have a cognitive reaction in their working memory to transform hazardous information into challenges or objectives [86]. However, hazard identification varies, according to the types of construction safety training received. The cognitive response formed by the workers who received safety training using two-dimensional drawings or pictures may not be the same as those that received the training using three-dimensional drawings, VR, or AR. The VR-based safety training program can provide a safe working environment where users can effectively practice electrical risk tasks, for example, improving their ability to cognize and intervene in electrical dangers [87]. For example, a construction worker trained using close-to-reality demonstrations may quickly identify hazards on-site. The essence of safety training is to assist construction workers in identifying job hazards while doing tasks on-site and avoiding them.

#### 3.1.3. Safety Knowledge Application

This stage is when construction workers apply the safety knowledge they have received on-site to prevent or avoid near-misses or accidents. Because of the complex nature of the construction sites, the stage takes place with reinforcement. The reinforcement includes site supervision to monitor safety violations of the construction workers and vice-versa. The reinforcement also consists of the primary (e.g., handrails and safety nets) and secondary preventive measures (e.g., PPE). Safety-abiding workers are rewarded accordingly in most organizations, and violators are punished. As explained in the previous section, the application of behaviorism theory in the construction industry concerns safety violations. Cognitive failures occur if, for instance, any construction worker fails to remember and apply what he was trained or remembers, but intentionally did not apply, which leads to a near-miss or accident on-site. However, there may be other factors that can cause cognitive failures that are beyond this study’s scope. The workers are expected to use the safety knowledge learned in the actual situation. Cognitive loss may be due to the kind of safety training a construction worker receives. It is assumed that those trained by advanced methods can quickly identify and avoid hazards on construction sites because of the fundamental nature of the training, and when they come across threats, they quickly use their working memory and prevent them, especially for more complex projects. The cognitive state of those who received advanced safety training methods may not be the same because the pictorial model stored in the long-term memory is not the same as that which is traditionally trained. However, the verbal model may be the same because it is assumed that they were all trained by safety and health professionals.

#### 3.1.4. Analysis, Synthesis, and Evaluation

These last three steps are beyond the scope of this study because they are for those who aspire to become experts in the safety and health profession in the construction industry. The analysis involves analyzing and interpreting the potential hazards or safety issues using only your expertise and safety knowledge to identify patterns and formulating theoretical explanations based on the observed phenomena. Synthesis is the process of planning, generating, and producing novel ways of hazard identification and other related safety issues. At this level, workers can contribute to developing the safety system. When confronted with a complex problem, people’s analytical inclination is to break it down into several sub-problems [85]. This stage and information comprehension are repeated until some new solutions are proposed. Evaluation is the capacity to assess the effectiveness of accident mitigation measures using predefined criteria and justifications. Typically, the acts are skill-based and require little mental effort. It is the most advanced degree of cognition. Once a construction worker reaches this stage, he may be called a safety professional. However, engineering knowledge is complex and varied, and mastering it requires sustained effort and concentrated training. Developing an expert-level understanding of a domain and its usage circumstances involves time, effort, and opportunity for practice with feedback. Individuals go through many phases to acquire the specialized knowledge associated with a particular cognitive skill. Individuals develop a skill far more quickly if they receive feedback on their actions. If they are mistaken, they must be informed of the nature of their error. It has long been established that practicing without feedback results in little learning.

### 3.2. Model Discussion and Implications

The model described in this article takes a cognitive approach to construction safety training and explains why the conventional safety training method may be inefficient and the advanced way may be more effective. Lack of or inadequate construction safety training is one of the primary reasons for workers’ unsafe behaviors. Obtaining, understanding, and applying relevant safety knowledge on-site are the core cognitive processes and failure to use those appropriately leads to near-misses or accidents. The cognitive model explains the process behind the failure of each cognitive link. Even though this describes the mechanisms of traditional and advanced construction safety training methods, the cognitive model is still a theoretical framework. The model’s propositions are based on problem solving, cognitive psychology, and cognitive learning theories. The study emphasized that the construction workers’ safety training may impact their mental state and accident occurrence. The rate of accidents on-site may be directly proportional to the type of safety training construction workers receive. The advanced safety training method may increase the mental state of the construction workers, and accidents on-site may be less likely to occur because of its advantages. Although most of the model’s concepts have been validated in psychology, education, and other relevant subjects, the model requires additional validation in construction.

Without safety vigilance, construction workers frequently adopt an unconscious and passive approach to searching for information about potential workplace risks [8]. However, this strategy can easily overlook information regarding potential workplace risks, resulting in cognitive failure to gather the appropriate safety knowledge. Workers with proper safety training and hazard identification ability develop specific cognitive responses to accurately estimate the magnitude of hazards and understand their features depending on the project’s complexity. Workers with inadequate training cannot identify potentially harmful circumstances on the job [22]. It is argued that workers have low hazard identification ability based on 2D drawings, pictures, or trainers’ experience, especially if the project is complicated. Conventional hazard recognition systems have not adequately addressed the issue of low-hazard recognition systems [23]. That is why innovative safety training is essential to eliminate the inefficiencies of the conventional method. The cognitive process starts with acquiring the proper safety training. If construction employees have the required safety training to detect potential hazards before the commencement of any project, near misses or accidents are less likely to occur.

A worker conceptualizes a situation to identify the information stored in long-term memory. The safety information may be obtained intentionally or unintentionally. The retrieval process disperses activation throughout significant long-term memory stores. Before accessing working memory, the information retrieved by purposeful search must pass through the external filter, which implies it must be received by sense organs (i.e., ears or eyes). However, impairments in the sense organs of workers and other site conditions (e.g., project complexity, hidden hazard, excessive noise) may result in failure. The working memory regularly generates calling circumstances that subconsciously pull associated knowledge from long-term memory to assist in danger analysis. Similarity matching and frequency gambling may fail if the calling conditions cannot access long-term memory information to analyze hazard information. Nonintentional searching lacks a search target, making it ineffective. This start mode may cause cognitive failures. However, it uses less attention than intentional searching, which is an advantage [1]. Both filters should filter nonintentional search results before accessing working memory. The internal filter should not obstruct information from the sense organs. Cognitive processes are constrained by working memory, attention, and relevance, which creates the internal filter. Nonintentional searching has a retrieval process; therefore, it may fail too.

The cognitive process advances to the second stage of information comprehension after the working memory has accumulated the relevant safety information. Construction employees’ difficulties are not as complex as those in nuclear power plants or aircraft operations [1]. They require only simple decisions and actions to deal with and avoid encountered hazards. Lack of safety training and job hazard identification comprehension may lead to cognitive failures. Workers trained with 3D BIM simulation demonstrated a greater awareness of safety training than those trained conventionally [24]. Cognitive failures occur if, for instance, any construction worker fails to remember and apply what he was taught, or remembers but intentionally did not, which leads to a near-miss or accident on-site. In addition, if construction workers lack the necessary professional skills and training, even if they adopt safe behaviour, there is a possibility that operational errors will lead to cognitive failure in action link formation. However, there may be other factors that can cause cognitive failures beyond this study’s scope. Safety perception is often due to the culture in which construction workers grew up and lived and the social acceptance of the dangers.

The impact of social influence on human behaviour and decision-making is significant. Numerous studies have discovered that workers choose unsafe actions, even when they know about a hazard and its possible dangers. On-site workers have a solid social structure, with workers eager to be accepted by others by demonstrating top-guy culture (not being scared of getting hurt) [15]. To avoid criticism, a worker may prevent a safe practice that is time-consuming and bothersome to coworkers and affects production, especially those who are newly employed. Unfortunately, construction is an industry where taking risks can have catastrophic results, including loss of life. Sometimes, safety criteria for a task are viewed as an impediment to completing the activity as quickly and effectively as possible [25]. Some personal protective equipment (PPE) is more cumbersome than helpful, but some believe that, if an activity requires specific PPE, it must be worn to complete the task safely [26]. The prevalent practice of paying employees based on price motivates employees to work as quickly as possible, without concern for safety or PPE, in order to earn the maximum daily money. Speed frequently requires cutting corners and accepting risks, so safety is commonly compromised. There is ongoing pressure to reach daily and weekly targets because of the looming deadlines for the completion of projects. The pressure is frequently felt most severely by site foremen, supervisors, and managers, who often turn a blind eye to harmful behaviours, as long as they meet the deadline [27]. Clients or owners will always prioritize figures and make decisions based on a comprehensive cost-benefit analysis when considering profitability [28]. On the other hand, site supervisors or managers frequently exert pressure on employees to work under a tight deadline and without PPE, resulting in the selection of risky behaviours.

Additionally, observations indicate that members of a closed social group (such as the construction industry) are resistant to external change. However, past studies have identified the primary aspects to consider while trying to penetrate a set of groups of people with the new technology. The critical factors of technology acceptance are perceived usefulness and perceived ease of use [29], prior usage and experience [30], self-efficacy [31], confidence in technology [32], subjective norm [33], expectations [34], user participation [35], risk [36], trust [37], gender and cultural diversity [38], technology features [39], attitude toward technology [40], and user perception [41]. Rogers et al. [42] stated that comparative advantage, compatibility, complexity, observability, and trialability determine technology spread. The outputs of technology are intended to enhance human capacities and expand human potential. The managers’ perception should be that using an innovative safety training method will improve performance and productivity and reduce near-misses or accidents. This can be achieved by demonstrating its advantages, such as a single integrated model, early warning signs, and monitoring machine, material, or worker locations, to mention a few, using an already built model. Share experiences of the tested models (i.e., the proposed advanced approach) with them to form intentions with the case study aid. If pre-existing knowledge matches new information, learning is enhanced. That could impact their beliefs and confidence in the new approach. By participating and evaluating the proposed method’s costs and benefits, they may accept or reject it, depending on how important they feel it is. The failure to meet users’ reasonable expectations has been cited as a major cause of unsuccessful system implementations. The assessed risks of the innovative safety training method could make the managers trust it, depending on how they understand the novel concept of advanced safety training. The comparative advantage, compatibility, complexity, observability, and trialability of the proposed method are also factors to examine before accepting any technology into full use. Once these critical factors are comprehensive, we believe that penetration into this closed group would be easier. Awareness of the benefits of open, collaborative efforts by project teams in the construction industry has led to a rise in the use of information technology [43]. However, the innovative approach may be much more attractive to young people than to long-term workers who already have developed mechanisms of conduct and response.

## 4. Conclusions

The mechanism models were built based on cognitive psychology and Bloom’s taxonomy six steps of cognitive learning theory. Construction workers’ safety training may be considered to focus on lower-order cognitive abilities, such as knowledge, comprehension, and application. The essence of the model is to explain the construction workers’ cognitive processes when they receive either the conventional or advanced safety training method. The cognitive process starts from the first step and ends where the safety knowledge is applied. A worker’s training plays a vital role in acquiring, storing, retrieving, and utilizing the appropriate information. If construction workers receive the proper safety training to identify potential hazards before the commencement of any project, near misses and accidents are less likely to occur. The knowledge is obtained from the external environment (safety trainers and 2D drawings, for example). It then goes to working memory before finally going to long-term memory (knowledge base)—the internal environment. Information is retrieved the same way it is stored, but, in this case, safety rules and potential hazards are the external environments. It is argued that the conventional safety training method may be inadequate because of its inefficiencies. Although it is not impossible to identify all potential hazards using the traditional way, it is easier and more efficient to use advanced techniques. However, the whole process may also depend on the construction worker’s experience and comprehension, the nature or complexity of the project, and other relevant factors. A worker might fail to correctly remember, interpret, and apply what he learned during training, based on the trainer’s experience and 2D drawings. It is assumed that those trained by advanced techniques can quickly identify and avoid hazards on construction sites because of the fundamental nature of the training, and when they come across threats, they quickly use their working memory and prevent them, especially for more complex projects. The cognitive state of those who received advanced safety training methods may not be the same because the pictorial model stored in the long-term memory is not the same as that which is traditionally trained. However, the verbal model may be the same because it is assumed they were all trained by safety professionals. Workers’ responses may not be the safest if they are influenced by production demands, coordination with coworkers, manager attitudes, and other relevant factors.

Some scholars concluded that a good safety training program improves workplace safety and health by preventing accidents [88,89]. Accident reduction on-site would improve construction productivity and company performance. The findings are expected to provide new insights for construction industry stakeholders about the shortcomings of the conventional safety training methods and the advantages of the advanced approach. This may help them adopt and utilize practical safety training to prevent or mitigate near-misses or on-site accidents. This study is probably the first to provide the mechanism models of the conventional and advanced methods of construction safety training based on cognitive psychology and cognitive learning theory. The findings of this study enhance the cognitive safety theory and aid in comprehending construction safety training procedures. This article contributes to the body of knowledge about construction safety training by expanding the authors’ understanding of the role of cognitive psychology and cognitive learning theory in the procedure. The main benefit of making such a model, from a cognitive point of view, is that it can help us learn more about the mental processes of two different types of construction safety training, and it can also help us come up with specific management suggestions to make up for the approaches’ flaws. The model contributes to research by providing a novel way to differentiate the two types of construction safety training using mental structure. Additionally, it presents a new approach rationale for how the nature of a worker’s safety training can result in a near-miss or deadly accident on-site. Future research will concentrate on the organizational aspects and other cognitive failures that could lead to near-misses or accidents on-site, based on the two types of safety training.

## Data Availability

Not applicable.
